# Supplementary material for: Analysis of gene expression profiles to study malaria vaccine dose efficacy and immune response modulation
Source: Genomics Inform. 2022 Sep 30;20(3):e32. doi: 10.5808/gi.22049 (PMC9576474; doi:10.5808/gi.22049)
Supplement: Supplementary Table 1. — Significantly expressed genes in male and female on control vs. controlled human malaria infection samples [file gi-22049suppl1.pdf]

Supplementary Table 1: Significantly expressed genes in male and female on control vs CHMI samples.

Ensembl ID	GeneSymbol
ENSG00000108700	CCL8
ENSG00000148053	NTRK2
ENSG00000151012	SLC7A11
ENSG00000160097	FNDC5
ENSG00000163879	DNALI1
ENSG00000184368	MAP7D2
ENSG00000197980	LEKR1
ENSG00000231535	LINC00278
ENSG00000233864	TTTY15
ENSG00000240184	PCDHGC3

Supplementary Table 2: Significantly expressed genes in protected and non-protected groups on control vs. CHMI samples.

Ensembl ID	GeneSymbol
ENSG00000074410	CA12
ENSG00000100065	CARD10
ENSG00000136944	LMX1B
ENSG00000145506	NKD2
ENSG00000151952	TMEM132D
ENSG00000162745	OLFML2B
ENSG00000163666	HESX1
ENSG00000169507	SLC38A11
ENSG00000182901	RGS7
ENSG00000197993	KEL
ENSG00000213934	HBG1
ENSG00000222414	RNU2-59P
ENSG00000224730	LILRB1-AS1
