# Supplementary material for: Analysis of gene expression profiles to study malaria vaccine dose efficacy and immune response modulation
Source: Genomics Inform. 2022 Sep 30;20(3):e32. doi: 10.5808/gi.22049 (PMC9576474; doi:10.5808/gi.22049)
Supplement: Supplementary Fig. 1. — Differential gene expression analysis using edgeR Pipeline. [file gi-22049suppl3.pdf]

Click "Ok" to move to next step

×

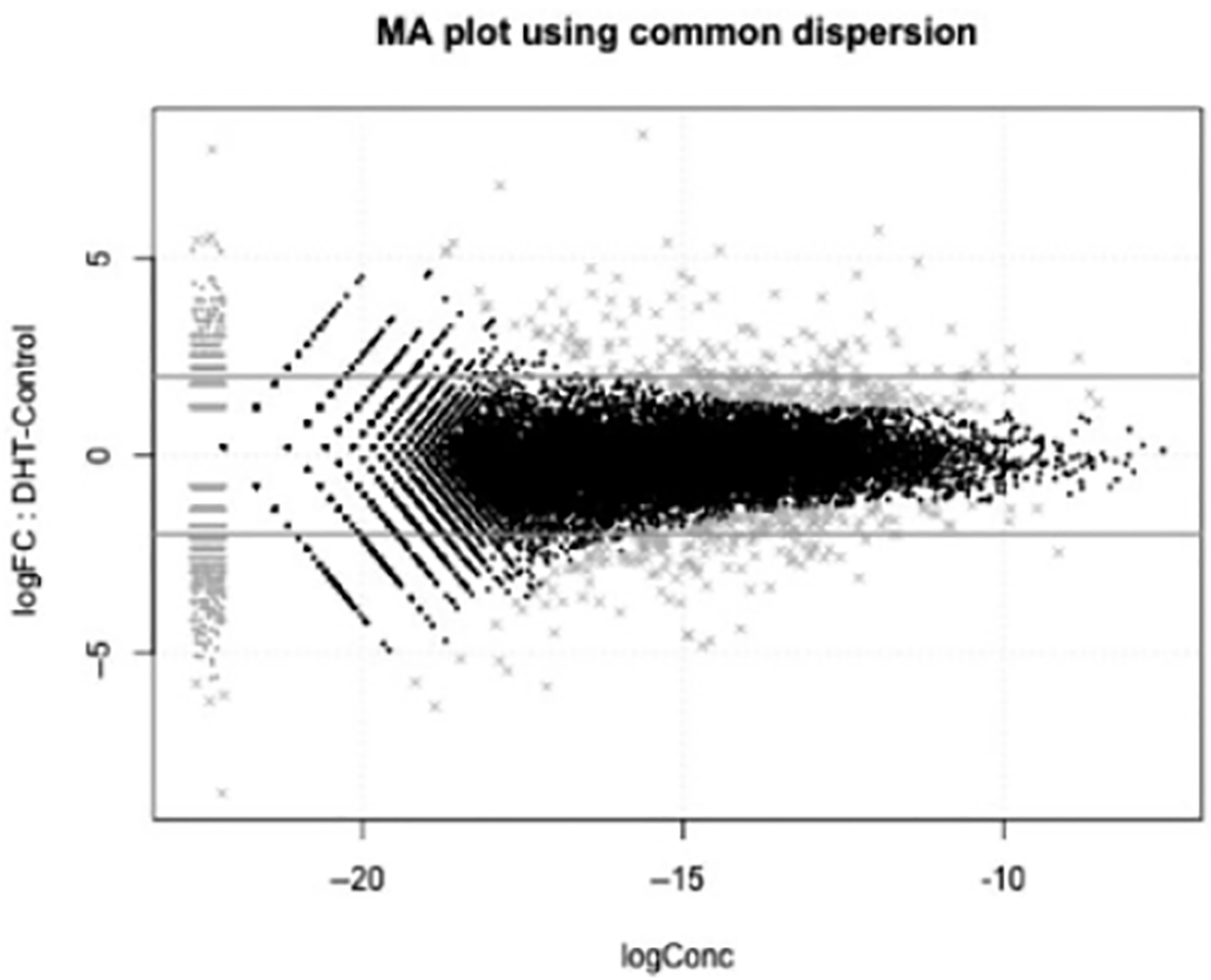

PARAMETERS

SAVE >

CANCEL

edgeR: differential expression between groups of replicates

edgeR is a Bioconductor software package for examining differential expression of replicated count data. An overdispersed Poisson model is used to account for both biological and technical variability. Empirical Bayes methods are used to moderate the degree of overdispersion across transcripts, improving the reliability of inference. The methodology can be used even with the most minimal levels
