# Supplementary figures and images for: Analysis of gene expression profiles to study malaria vaccine dose efficacy and immune response modulation
Source: Genomics Inform. 2022 Sep 30;20(3):e32. doi: 10.5808/gi.22049 (PMC9576474; doi:10.5808/gi.22049)

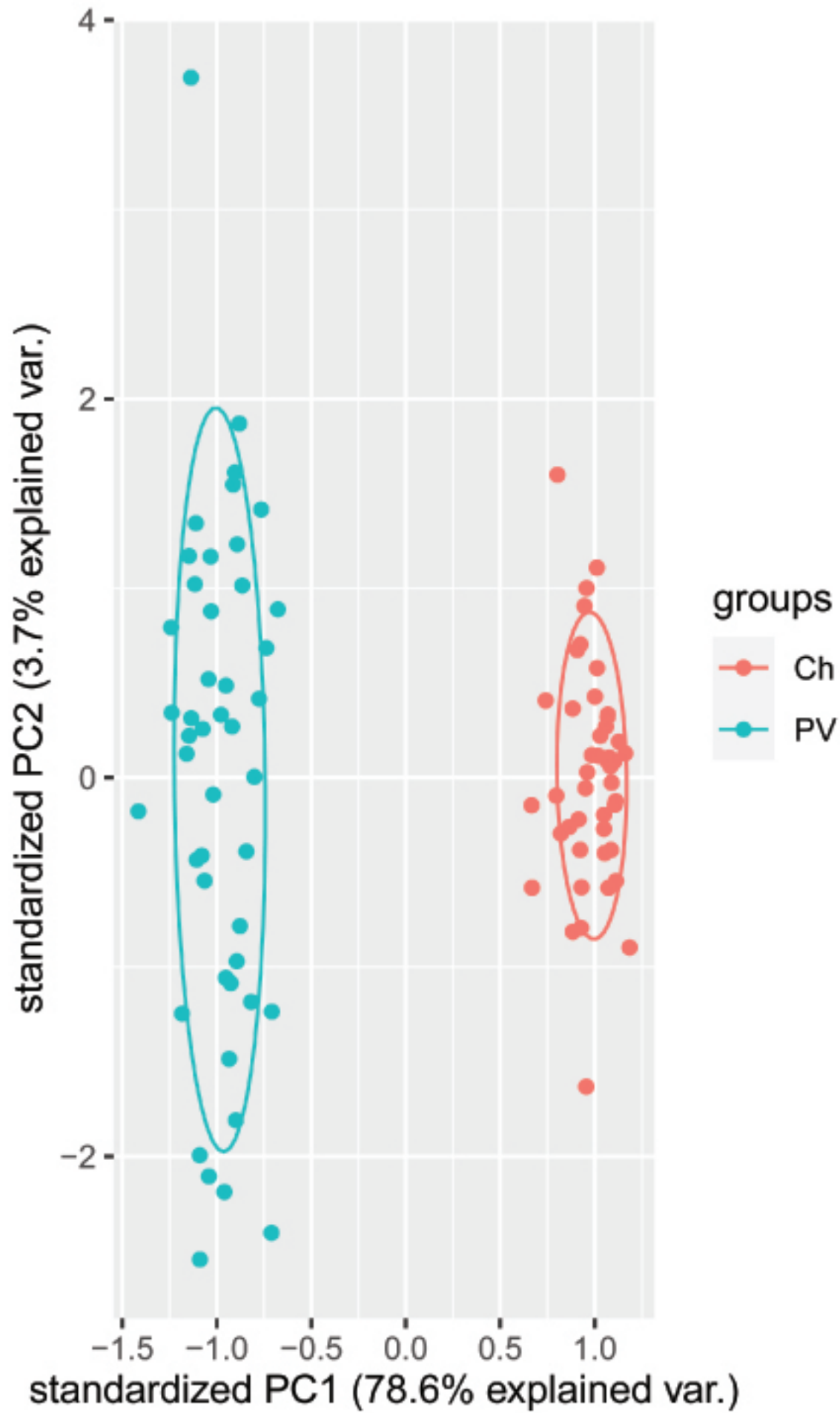

Supplement: Supplementary Fig. 3. — Principal component analysis plot for control vs. controlled human malaria infection samples for significant genes. [file gi-22049suppl5.pdf]

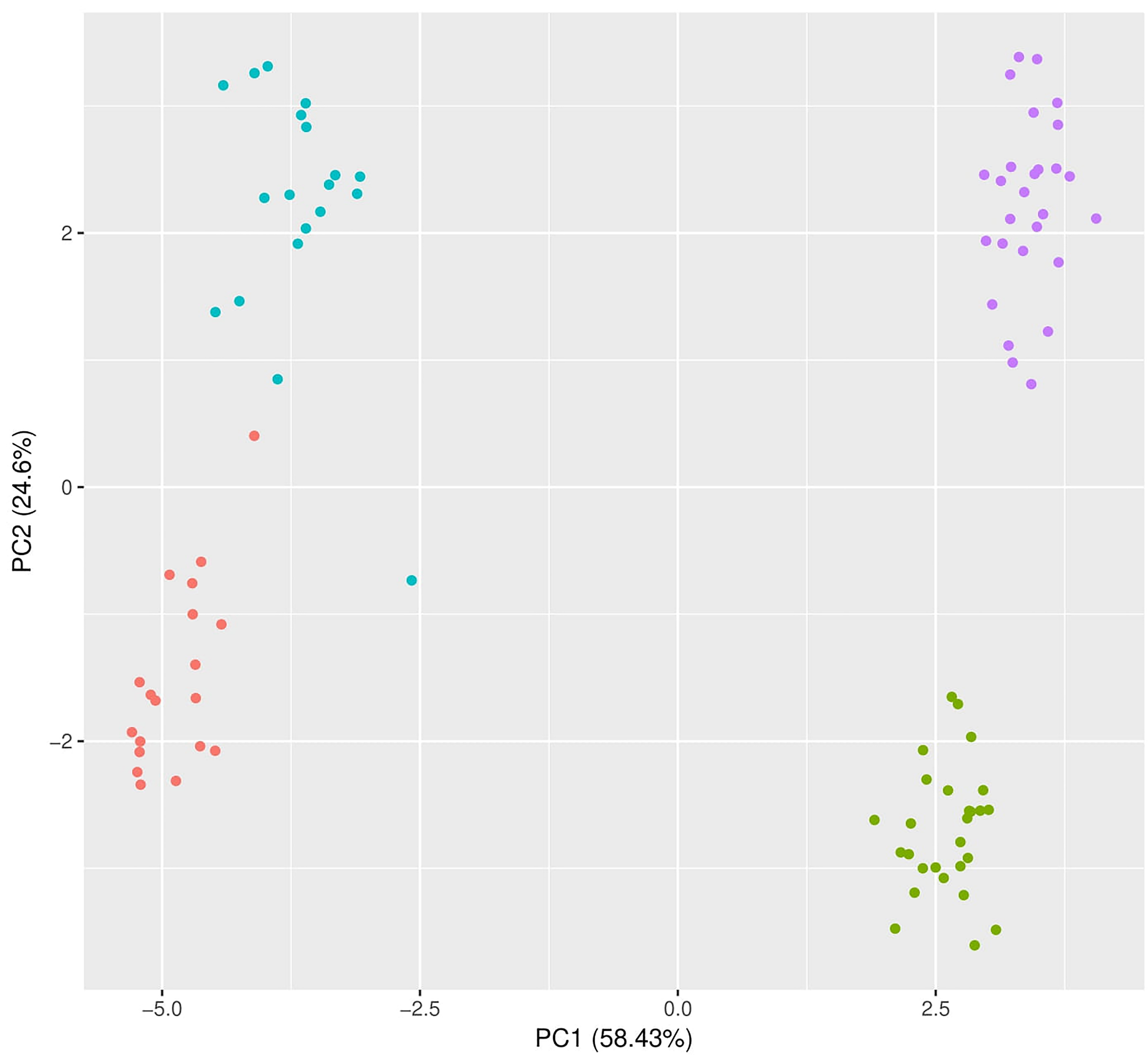

Supplement: Supplementary Fig. 4. — Significantly expressed genes in males and females on control vs. controlled human malaria infection samples. [file gi-22049suppl6.pdf]

# JAK-STAT SIGNALING PATHWAY

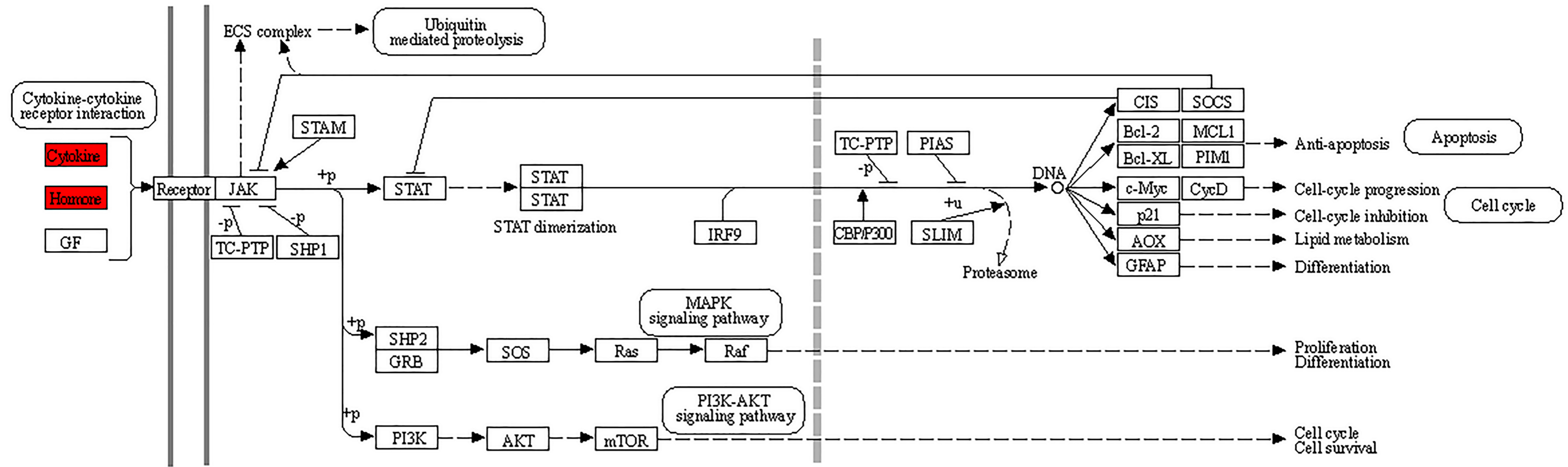

Supplement: Supplementary Fig. 5. — JAK-STAT pathway. [file gi-22049suppl7.pdf]

# PATHWAYS IN CANCER

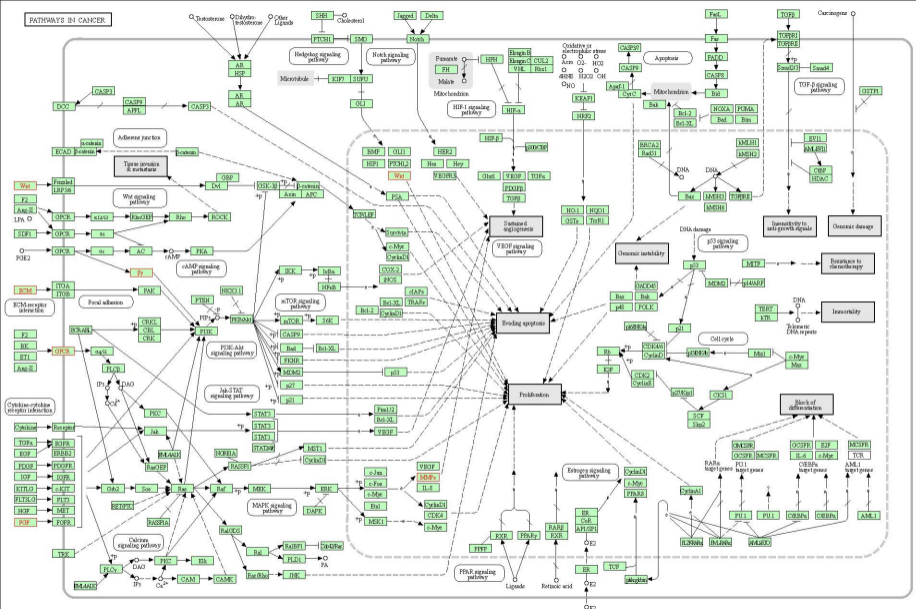

Supplement: Supplementary Fig. 6. — Pathways in cancer. [file gi-22049suppl8.pdf]

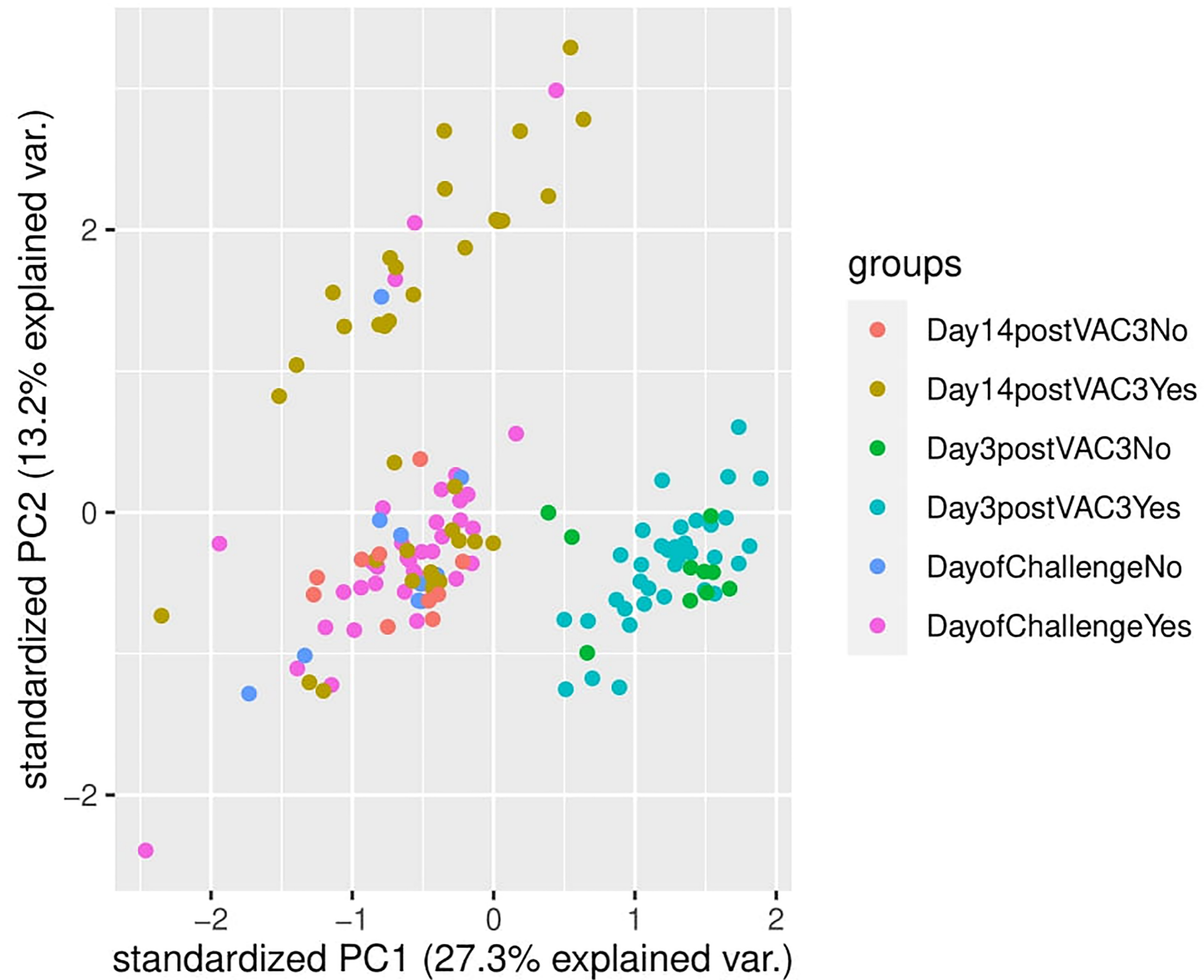

Supplement: Supplementary Fig. 7. — Principal component analysis plot of protected and non-protected samples on various days. [file gi-22049suppl9.pdf]
