# Supplementary material for: Analysis of gene expression profiles to study malaria vaccine dose efficacy and immune response modulation
Source: Genomics Inform. 2022 Sep 30;20(3):e32. doi: 10.5808/gi.22049 (PMC9576474; doi:10.5808/gi.22049)

PIPELINE INPUT

RUN PIPELINE

Click “Upload files” and chose file that should be analyzed. When data are successfully uploaded, click “Continue” and “Start”

FACTOR ANALYSIS

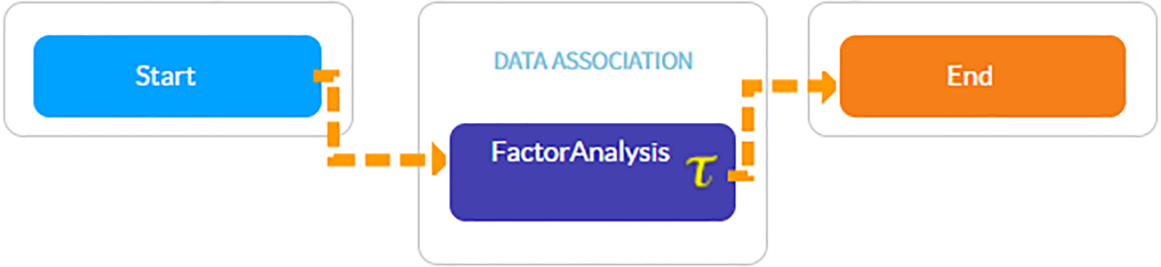

Supplement: Supplementary Fig. 8. — Quantile normalization pipeline. [file gi-22049suppl10.pdf]
